# Beyond the Vestibulo-Ocular Reflex: Vestibular Input is Processed Centrally to Achieve Visual Stability

**DOI:** 10.3390/vision2020016

**Published:** 2018-03-21

**Authors:** Edwin S. Dalmaijer

**Affiliations:** MRC Cognition and Brain Sciences Unit, University of Cambridge, Cambridge CB2 1TN, UK; edwin.dalmaijer@mrc-cbu.cam.ac.uk; Tel.: +44(0)-1223-355-294

**Keywords:** visual stability, galvanic vestibular stimulation, visual tilt, ocular torsion, multi-sensory integration

## Abstract

The current study presents a re-analysis of data from Zink et al. (1998, *Electroencephalography and Clinical Neurophysiology*, *107*), who administered galvanic vestibular stimulation through unipolar direct current. They placed electrodes on each mastoid and applied either right or left anodal stimulation. Ocular torsion and visual tilt were measured under different stimulation intensities. New modelling introduced here demonstrates that directly proportional linear models fit reasonably well with the relationship between vestibular input and visual tilt, but not to that between vestibular input and ocular torsion. Instead, an exponential model characterised by a decreasing slope and an asymptote fitted best. These results demonstrate that in the results presented by Zink et al. (1998), ocular torsion could not completely account for visual tilt. This suggests that vestibular input is processed centrally to stabilise vision when ocular torsion is insufficient. Potential mechanisms and seemingly conflicting literature are discussed.

## 1. Introduction

**Figure 1:**
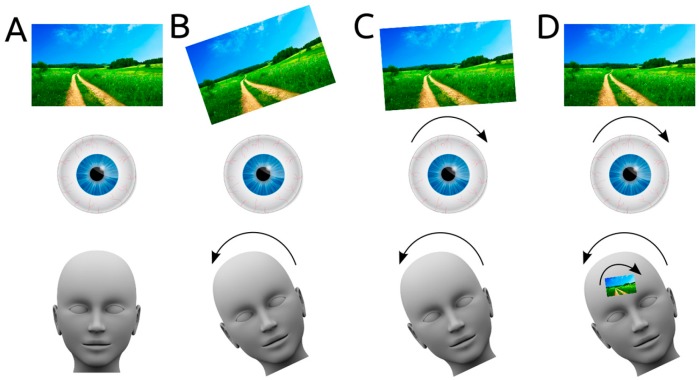
(**A**) When the head is in an upright position, so are the eyes and the visual world. (**B**) If the eyes were to remain upright with respect to a rolling head, the visual world would tilt. (**C**) Due to the vestibulo-ocular reflex, the eyes rotate in the opposite direction of the rolling head. This counteracts most of the rotation of the visual field. (**D**) Some authors have argued that vestibular input is also processed centrally, to directly tilt visual fields.

During everyday movements like walking, the human head and eyes continuously move, yet humans have a relatively stable visual perception of the world. This is due to the vestibulo-ocular reflex. When the vestibular system senses a head movement, it signals directly to the eye muscles, and a compensatory eye movement is produced to realign the visual world (Figure 1A–C). Specifically, when the human head makes a rolling movement, a compensatory torsional eye movement in the opposite direction is generated, thereby keeping the visual field stable.

However, torsional eye movements induced by the vestibulo-ocular reflex can be prevented by fixating the eyeballs of anaesthetised cats to a metal ring. In these cats, the receptive fields of a proportion of neurons in the visual cortex tilted when the cat’s head was tilted (roll motion), compared to when it was in an upright position [1,2,3]. These results suggest that another mechanism might exist to centrally process vestibular information when the vestibulo-ocular reflex is disrupted (Figure 1D).

This suggestion is not without controversy: Receptive fields did not tilt as a function of head roll in cats that were not anaesthetised and did not have their eyeballs fixated [4]. In those cats, receptive field tilt did occur, but it was uncorrelated with head roll. It was thus suggested that the tilting of receptive fields could have been an effect of non-specific arousal (with the head movement as an arousing stimulus), or could be interpreted as “broadening of direction preference”. Therefore, it remains unclear whether central processing of vestibular input influences visual stability beyond its role in the vestibulo-ocular reflex.

Ideally, the hypothesised central processing of vestibular information could be directly tested by comparing the effects of vestibular input on visual tilt and on ocular torsion. If the vestibulo-ocular reflex is sufficient for stabilising the visual world, the ocular torsion induced by vestibular input should be linearly related to the induced visual tilt. Such a study exists: Zink and colleagues reported that both ocular torsion and visual tilt increase with vestibular input [5].

Human research participants do not normally have their eyes fixated to metal rings while their body is tilted. Instead, Zink and colleagues induced vestibular input by means of galvanic vestibular stimulation. This technique has been known for over a hundred years [6], with the first reports dating back to around 1900 [7,8]. Galvanic vestibular stimulation is an electric current (usually applied via both mastoids) that stimulates the vestibular neuronal afferents and is known to induce spontaneous nystagmus (at intensities over 3 mA) and ocular torsion [5,9,10].

Eye movements induced by galvanic vestibular stimulation occur due to electrical stimulation of semicircular canal afferents [11]. Although otolith activation occurs at higher stimulation intensity, its contribution to eye movement varies between individuals [5,9]. A contemporary model on the contribution of semicircular canal and otolith activation is provided by Day and colleagues [12], who built on earlier models by the same group [13] and others [10].

In the aforementioned study by Zink and colleagues, it was reported that both ocular torsion and visual tilt increase with galvanic vestibular stimulation at higher current intensities [5]. Importantly, induced ocular torsion would have invoked visual tilt merely due to the rotation of both eyeballs. However, from the analysis by Zink and colleagues, it is unclear whether ocular torsion could account for all the induced visual tilt, or whether part of the visual tilt could have been ascribed to the central processing of the vestibular input.

The current study aims to investigate the relationship between ocular torsion and visual tilt through re-analysing the results of Zink and colleagues by modelling the contributions of vestibular stimulation on ocular torsion and visual tilt. Specifically, the models introduced here take into account a hypothesised limit on ocular torsion. If such a limit exists, visual tilt will increasingly depend on central processing when vestibular input increases. Alternatively, if both ocular torsion and visual tilt are linearly related to vestibular input, it would suggest the vestibulo-ocular reflex is sufficient, and no central processing of vestibular input is required to stabilise vision.

## 2. Materials and Methods 

### 2.1. Zink et al. (1998)

Relevant data was extracted from Zink and colleagues [5], who conveniently provided it in tables with descriptive statistics of both visual tilt and ocular torsion as a function of vestibular stimulation intensity (reproduced in Table 1). They used electrodes taped to both mastoids to deliver a unipolar direct current. During a trial, the polarity and intensity of the stimulation was kept constant, but they could be varied between trials. Specifically, Zink and colleagues tested both left-anodal and right-anodal stimulation, and they applied current intensities between 1 and 7 mA. Not all participants received all current intensities, presumably because the stimulation caused discomfort or pain. The numbers of participants in each cell of Zink and colleagues’ design are reported in Table 1.

Stimulation trials lasted for 5 s, during which static ocular torsion was measured using a laser-scanning opthalmoscope that recorded the fundus in both eyes on video.

To measure visual tilt, participants were positioned in front of a half-open dome with a diameter of 60 cm that completely covered their visual field and was covered with a pattern of randomly placed coloured dots. The dome prevented participants from using straight lines in the environment as references. Perceived visual tilt was measured using a centrally presented line that participants could adjust to the level of rotation that they perceived during galvanic vestibular stimulation.

Ocular torsion occurred towards the anode (counter-clockwise under left anodal stimulation, clockwise in right anodal stimulation). Visual tilt occurred away from the anode (clockwise in left anodal stimulation and counter-clockwise in right anodal stimulation). The results from Zink and colleagues are reproduced in Table 1.

For all analyses presented here, the left and right anodal stimulation was averaged within each stimulation intensity. One measurement that was inconsistently reported by Zink and colleagues has been omitted from the current study. Specifically, Zink and colleagues refer to a measurement of visual tilt at a stimulation intensity of 4.5 mA. However, their visual tilt plot includes a point at 3.5 mA, and the point in question does not appear in their visual tilt table.

### 2.2. Linear Models

Zink and colleagues fit linear models that describe both ocular torsion and visual tilt as a function of stimulation intensity (Equations (1) and (2)). Free variables *a* and *b* in these equations determine the slope and intercept of the function. It should also be noted that the *a* and *b* parameters in the ocular torsion equation are independent from those in the visual tilt equation.

(1) $$T_{ocular}=a_{o}\cdot V+b_{o}$$

(2) $$T_{visual}=a_{v}\cdot V+b_{v}$$

in which *T_ocular_* is ocular torsion in degrees, *V* is vestibular input in mA, *a_o_* is the slope in degrees per mA, and *b_o_* the intercept in degrees in the relationship between ocular torsion and visual tilt. *T_visual_* is visual tilt in degrees, *a_v_* is the slope in degrees per mA, and *b_v_* is the intercept in degrees.

### 2.3. Directly Proportional Linear Models

By accounting for an intercept in Equations (1) and (2), Zink and colleagues allowed the baseline ocular torsion to be different from 0 degrees at 0 mA of stimulation. Because stimulation was applied in two directions, according to Equation (1) the eye is in a different baseline position depending on whether −0 or 0 mA of stimulation is applied. The same is true for Equation (2) and visual tilt. In other words, a non-zero a_o_ parameter in Equation (1) means that the eye is in two different orientations at the same time when no stimulation is applied. The same is true for the a_v_ parameter in Equation (2): non-zero values would mean visual perception will be tilted in two directions at the same time when no stimulation is applied.

Evidently, this cannot be true: At 0 mA of stimulation, the eye and visual field should be un-rotated. The best way to account for this is by requiring that vestibular input and ocular torsion (or visual tilt) be directly proportional. This idea was implemented in Equations (3) and (4), which both have only one free variable that determines the slope of the function.

(3) $$T_{ocular}=a_{o}\cdot V$$

(4) $$T_{visual}=a_{v}\cdot V$$

in which *T_ocular_* is ocular torsion in degrees, *V* is vestibular input in mA, and *a_o_* is the slope in degrees per mA in the relationship between ocular torsion and visual tilt. *T_visual_* is visual tilt in degrees, and *a_v_* is the slope in degrees per mA in the relationship between visual tilt and vestibular input.

### 2.4. Exponential Model of Ocular Torsion and Resulting Visual Tilt

If ocular torsion is indeed limited by an upper bound, neither linear model described above would describe the relationship between galvanic vestibular stimulation and ocular torsion accurately. Instead, as vestibular input increases, the slope of the increase in ocular torsion is expected to decrease and to become 0 when an asymptote is reached. Equation (5) describes such a relationship, in which at high levels of vestibular input, ocular torsion will be no higher than asymptote *b*.

If perceived visual tilt is solely a product of ocular torsion, its relationship with vestibular input should be the same as that of ocular torsion, as described by Equation (6).

(5) $$T_{ocular}=b_{o}\cdot \left(1-e^{-V/a_{o}}\right)$$

(6) $$T_{visual}=b_{v}\cdot \left(1-e^{-V/a_{v}}\right)$$

in which *T_ocular_* is the ocular torsion in degrees, *V* is the vestibular input in mA, *a_o_* determines the slope of the function (with lower numbers reflecting a steeper slope), and *b_o_* determines the asymptote of the function (preventing *T_ocular_* to ever rise above *b_o_*, regardless of the value of *V*). *T_visual_* is the visual tilt in degrees, and a_v_ and *b_v_* have the same purpose as a_o_ and b_o,_ but can be of different values.

### 2.5. Curve Fitting

To fit Equations (1)–(6) to the data reported by Zink and colleagues (reproduced in Table 1), the unsigned results from left-anodal and right-anodal stimulation were first averaged. Then, using least squares estimation in a full exploration of parameter space, the optimal combination of parameter values were assessed for the three types of models outlined above (linear, directly proportional linear, and exponential) for ocular torsion and visual tilt independently.

Fitting was performed in custom Python scripts [14,15], using NumPy [16] for computations and Matplotlib [17] for plotting. This code is available from GitHub on https://github.com/esdalmaijer/zink_et_al_1998_re-analysis.

## 3. Results

### 3.1. Parameter Estimates

Parameter space was explored within the range 0–3.5 with a grid resolution of 0.0005 for parameters *a* and *b* in linear models (Equations (1)–(4)). The space was explored within the range 0–7 with grid resolution 0.001 for parameters *a* and *b* in exponential models (Equations (5) and (6)).

**Figure 2:**
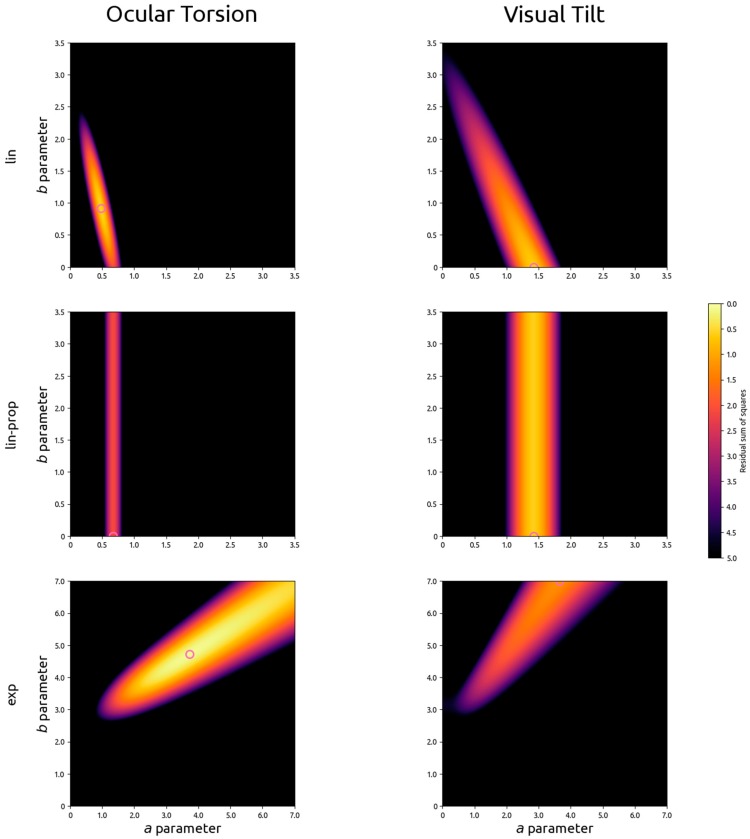
Visualisation of the residual sum of squares in parameter space as a function of the a (*x*-axis) and b parameter (*y*-axis) in linear models (Equations (1) and (2); top row, titled ‘lin’), directly proportional linear models (Equations (3) and (4); middle row, titled ‘lin-prop’), and exponential models (Equations (5) and (6); bottom row, titled ‘exp’) of the relationship between galvanic vestibular stimulation and ocular torsion (**left column**) or visual tilt (**right column**). Lower values indicate better fits and are indicated by lighter colours. The best fit is indicated by a pink circle.

Parameter estimates are visualised in Figure 2, with the best fitting combination of parameters indicated with a circle. The best fits for ocular torsion were *a_o_* = 0.483 and *b_o_* = 0.913 for the linear model (Equation (1)), *a_o_* = 0.672 for the directly proportional linear model (Equation (3)), and *a_o_* = 3.722 and *b_o_* = 4.714 for the exponential model (Equation (5)). The best fits for visual tilt were *a_v_* = 1.421 and *b_v_* = 0.000 for the linear model (Equation (2)), *a_v_* = 1.421 in the directly proportional linear model (Equation (4)), and *a_v_* = 3.649 and *b_v_* = 7.00 for the exponential model (Equation (6)).

### 3.2. Ocular Torsion

**Figure 3:**
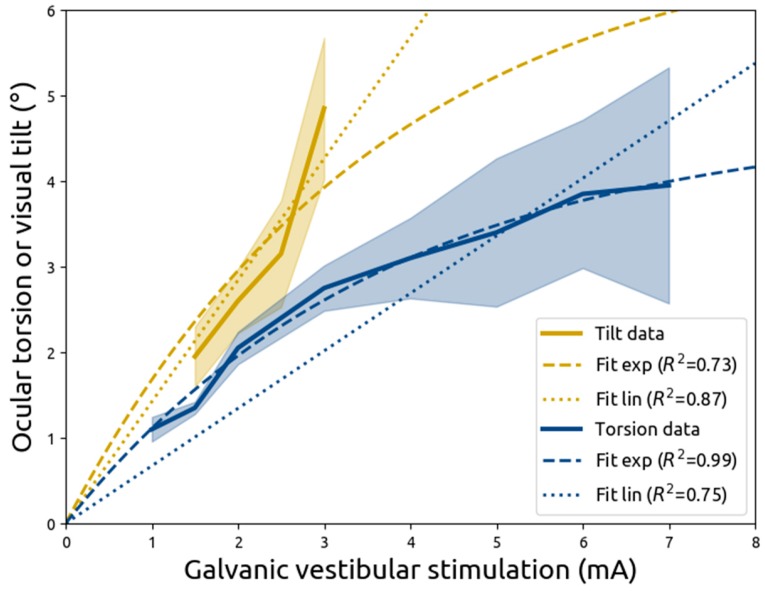
Ocular torsion (blue) and visual tilt (yellow) in degrees (*y*-axis) as a function of unipolar direct current galvanic vestibular stimulation (*x*-axis). Solid lines represent the average and shading the standard error of the mean in data reported by Zink et al. (1998). Dotted lines represents directly proportional linear fits (Equations (3) and (4)), and dashed lines represent exponential model fits (Equations (5) and (6)).

A directly proportional linear model (Equation (3)) explained 75 percent of the variance in ocular torsion under galvanic vestibular stimulation (Figure 3, in blue). By contrast, an exponential model (Equation (5)) explained 99 percent of the variance.

A linear model with a free intercept variable (Equation (1)) explained 94 percent of the variance but is biologically impossible due to its baseline position of 0.913 degrees of ocular rotation at 0 mA of galvanic vestibular stimulation and −0.913 degrees at −0 mA.

### 3.3. Visual Tilt

For visual tilt under galvanic vestibular stimulation (Figure 3, in yellow), a directly proportional model (Equation (4)) explained 87 percent of the variance and an exponential model (Equation (6)) explained 73 percent.

A linear model with a free intercept variable (Equation (2)) accounted for the same amount of variance as the directly proportional linear model, because the best fitting parameters were equal for both (a slope of 1.42 degrees per mA, and an intercept of 0).

### 3.4. Visual Tilt as a Function of Ocular Torsion

**Figure 4:**
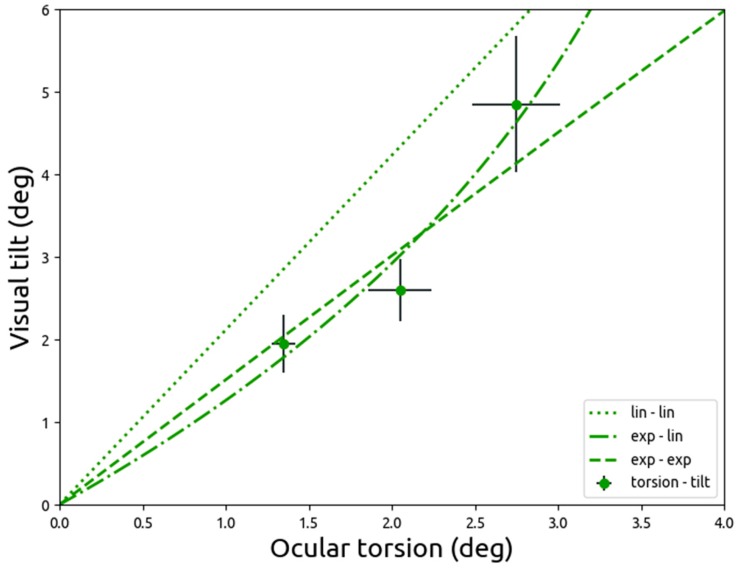
The relationship between the effects of galvanic vestibular stimulation on ocular torsion (*x*-axis) and visual tilt (*y*-axis). Points indicate averages of data reported by Zink et al. (1998) for galvanic vestibular stimulation unipolar direct current intensities 1.5, 2.0, and 3.0 mA, and error bars indicate the standard error of the mean. The dotted line (labelled ‘lin–lin’) is a combination of directly proportional linear models of the relationship between vestibular input and ocular torsion (Equation (3)) or visual tilt (Equation (4)). The dashed line (labelled ‘exp–exp’ is a combination of exponential models of the relationship between vestibular input and ocular torsion (Equation (5)) or visual tilt (Equation (6)). The dashed-dotted line (labelled ‘exp–lin’) represents a combination of Equations (4) and (5). The fits are the same as those presented in Figure 2 and Figure 3; they are simply replotted in the same space.

When visual tilt is plotted as a function of ocular torsion under the same galvanic vestibular stimulation intensities (Figure 4), it is best fitted by a combination of an exponential model of the relationship between galvanic vestibular stimulation and ocular torsion (Equation (5)) and a directly proportional linear model of the relationship between galvanic vestibular stimulation and visual tilt (Equation (4)). The relationship between ocular torsion and visual tilt under galvanic vestibular stimulation is worst fitted by a combination of directly proportional linear models of both (Equations (3) and (4)). Exponential models of both (Equations (5) and (6)) also fit sub-optimally.

## 4. Discussion

In the current study, data from Zink and colleagues (1998) was reanalysed using models that more accurately reflected biology, specifically the non-tilted position of the eye at no vestibular input. Zink and colleagues applied galvanic vestibular stimulation, using a unipolar and direct current, and measured either ocular torsion or visual tilt. In the current study, it is demonstrated that a directly proportional linear model fits the data from both measures relatively well. However, ocular torsion is better described by a model with an exponentially decreasing slope that moves towards an asymptote. These results show that with increasing vestibular stimulation, ocular torsion slopes down, whereas visual tilt keeps increasing linearly. The lack of a linear relationship between the vestibular effects on ocular torsion and on visual tilt suggests that ocular torsion is not the sole contributor of visual tilt, but that instead vestibular input could be processed centrally to maintain visual stability.

### 4.1. Central Processing of Vestibular Information

In multi-sensory research, the most simple explanation for the interaction between two senses (e.g., the vestibular and visual system) is often stochastic resonance [18]. Stochastic resonance occurs when the general level of activation of multi-sensory neurons is heightened by a stimulus (e.g., galvanic vestibular stimulation or auditory noise), which leads to a higher sensitivity to faint stimuli from another sense (e.g., tactile information) that on its own would lead to sub-threshold activation for detection. An example of such a study is by Ferrè and colleagues [19], who showed that galvanic vestibular stimulation indeed leads to a higher sensitivity for a faint tactile stimulus. They offer an explanation based in stochastic resonance, hypothesising simultaneous activation of bimodal neurons in parietal opperculum. However, the data from Zink and colleagues re-analysed here cannot be explained by stochastic resonance, as the observed visual tilt was direction-dependent on the stimulation’s current direction.

An alternative explanation is that vestibular information is processed in the visual cortex. When the heads of anaesthetised cats with fixated eyeballs are rolled, the receptive fields of a proportion of neurons in their visual cortex tilt as a result [1,2,3]. This suggests that a mechanism might exist by which head roll that is not otherwise compensated for is compensated for by the rotation of receptive fields.

Contrary to the above, non-anaesthetised cats with non-fixated eyeballs do show tilt in receptive fields in a proportion of neurons in visual cortex, but they are uncorrelated with head roll [4]. It was suggested that receptive field tilting was a response to general arousal rather than a systematic processing of visual tilt.

### 4.2. Alternative Explanations for a Non-Linear Relationship between Vestibular Input and Ocular Torsion

Although Zink and colleagues used direct current stimulation, others have employed vestibular stimulation with a sinusoidally alternating current and have instead argued that visual tilt can be completely accounted for by ocular torsion [20]. In addition, using 100 ms pulses, Aw and colleagues found a linear relationship between current intensity and the velocity of ocular torsion [21]. In sum, a linear relationship between vestibular input and ocular torsion does exist when pulsed or alternating current galvanic vestibular stimulation is employed.

A direct investigation of the non-linear properties of the torsional response to natural vestibular stimulation (by means of head rotation) is described by Schneider and colleagues, who modelled the gain and intensity of torsional nystagmus [22]. Specifically, they demonstrate that nystagmus was present during low-frequency (such as Zink and colleagues’ stimulation), but not high-frequency, stimulation, and they argue that it is the contribution of nystagmus that causes the non-linear relationship between vestibular input and ocular torsion. In the mechanism that Schneider and colleagues propose, vestibular input induces nystagmus, which is in turn centrally processed. This process in turn interferes with the vestibularly induced generation of ocular torsion, resulting in a non-linear relationship between vestibular input and ocular torsion.

### 4.3. Vestibular Effects on Attention

Another potentially interesting role of vestibular input is highlighted by Shuren and colleagues, who rotated participants in a revolving chair before asking them to perform a line bisection task. After leftward rotation, participants showed an increased leftward bisection error, suggesting that vestibular input might induce an attentional bias [23].

Further evidence for a potential role of vestibular functioning in spatial attention is provided by studies of vestibular stimulation in neglect syndrome. Neglect syndrome occurs primarily after damage to right parietal cortex, and patients display a strong attentional bias towards ipsilesional space. Neglect patients but not control patients (with similar lesions but without neglect) show deviations of their perceived visual vertical during roll head movements [24], and exhibit a larger deviation of subjective visual vertical and horizontal [25]. Furthermore, neglect symptoms decrease during [26] or immediately after caloric vestibular stimulation, but these effects do not linger for more than 15 min [27].

## 5. Conclusions 

Vestibular input by means of galvanic vestibular stimulation leads to both ocular torsion as a result of the vestibulo-ocular reflex and visual tilt as a result of ocular torsion. However, visual tilt is not always linearly related to ocular torsion under galvanic vestibular stimulation, hinting at an additional central processing of vestibular information to stabilise vision. One such mechanism could be the tilting of receptive fields in visual cortex, although results supporting this theory are inconsistent. Alternatively, it has been suggested that low-frequency vestibular stimulation induces a nystagmus that interferes with the generation of ocular torsion and thus results in a non-linear relationship between vestibular input and ocular torsion. In conclusion, central processing of vestibular information and induced nystagmus occurs to stabilise vision during rolling head movements.

## Figures and Tables

**Table 1:** Results from Zink et al. (1998), *Electroencephalography and Clinical Neurophysiology*, *107*, pp. 200–205. Zink and colleagues applied unipolar direct current with the anode on the right or left mastoid. They measured ocular torsion and visual tilt in degrees of rotation at different galvanic vestibular stimulation intensities. Results reported by Zink and colleagues (and reprinted here) are the average rotation (unsigned), the standard deviation (between round brackets), the minimum and maximum measured values (between square brackets), and the number of participants tested in a particular cell. Ocular torsion occurred towards the anode, whereas visual tilt occurred away from the anode.

Current Strength	Left Anodal Stimulation		Right Anodal Stimulation	
(mA)	Ocular Torsion	Visual Tilt	Ocular Torsion	Visual Tilt
1.0	1.0 (0.4) [0.5–1.5] *N* = 6		1.2 (0.3) [0.6–1.4] *N* = 6	
1.5	1.3 (0.1) [1.2–1.4] *N* = 2	2.2 (0.9) [1.3–3.3] *N* = 4	1.4 (0.1) [1.3–1.4] *N* = 2	1.7 (0.5) [1.3–2.3] *N* = 4
2.0	2.0 (0.5) [1.3–2.5] *N* = 7	2.6 (1.4) [1.3–6.3] *N* = 12	2.1 (0.5) [1.5–2.8] *N* = 7	2.6 (1.2) [1.0–5.8] *N* = 12
2.5		3.2 (2.3) [1.2–9.4] *N* = 12		3.1 (2.0) [1.0–8.5] *N* = 12
3.0	2.5 (0.8) [1.4–3.5] *N* = 7	4.9 (1.5) [3.0–6.5] *N* = 4	3.0 (0.6) [2.2–3.5] *N* = 7	4.8 (1.8) [2.6–6.4] *N* = 4
4.0	2.9 (1.0) [1.2–3.8] *N* = 6		3.3 (1.3) [1.3–4.2] *N* = 6	
5.0	3.2 (1.1) [2.0–4.1] *N* = 3		3.6 (1.9) [1.5–4.3] *N* = 3	
6.0	3.6 (1.3) [2.2–4.5] *N* = 3		4.1 (1.7) [2.2–5.2] *N* = 3	
7.0	3.9 (1.8) [2.6–5.2] *N* = 2		4.0 (2.1) [2.5–5.4] *N* = 2	

## References

[1] Denney D., Adorjanti C. (1972). Orientation specificity of visual cortical neurons after head tilt. Exp. Brain Res..

[2] Horn G., Hill R.M. (1969). Modifications of Receptive Fields of Cells in the Visual Cortex occurring Spontaneously and associated with Bodily Tilt. Nature.

[3] Horn G., Stechler G., Hill R.M. (1972). Receptive fields of units in the visual cortex of the cat in the presence and absence of bodily tilt. Exp. Brain Res..

[4] Schwartzkroin P.A. (1972). The effect of body tilt on the directionality of units in cat visual cortex. Exp. Neurol..

[5] Zink R., Bucher S.F., Weiss A., Brandt T., Dieterich M. (1998). Effects of galvanic vestibular stimulation on otolithic and semicircular canal eye movements and perceived vertical. Electroencephalogr. Clin. Neurophysiol..

[6] Day B.L. (1999). Galvanic vestibular stimulation: New uses for an old tool. J. Physiol..

[7] Buys E. (1909). Beitrag zum Studium des Galvanischen Nystagmus mit Hilfe der Nystagmographie. Mschr Ohrenheilk.

[8] Hitzig E. (1898). Der Schwindel (Vertigo).

[9] Kleine J.F., Guldin W.O., Clarke A.H. (1999). Variable otolith contribution to the galvanically induced vestibulo-ocular reflex. NeuroReport.

[10] Schneider E., Glasauer S., Dieterich M. (2000). Central processing of human ocular torsion analyzed by galvanic vestibular stimulation. NeuroReport.

[11] Schneider E., Glasauer S., Dieterich M. (2002). Comparison of Human Ocular Torsion Patterns During Natural and Galvanic Vestibular Stimulation. J. Neurophysiol..

[12] Day B.L., Ramsay E., Welgampola M.S., Fitzpatrick R. (2011). The human semicircular canal model of galvanic vestibular stimulation. Exp. Brain Res..

[13] Fitzpatrick R., Day B.L. (2004). Probing the human vestibular system with galvanic stimulation. J. Appl. Physiol..

[14] Dalmaijer E.S. (2017). Python for Experimental Psychologists.

[15] Van Rossum G., Drake F.L. (2011). Python Language Reference Manual.

[16] Oliphant T.E. (2007). Python for Scientific Computing. Comput. Sci. Eng..

[17] Hunter J.D. (2007). Matplotlib: A 2D Graphics Environment. Comput. Sci. Eng..

[18] Lugo E., Doti R., Faubert J. (2008). Ubiquitous Crossmodal Stochastic Resonance in Humans: Auditory Noise Facilitates Tactile, Visual and Proprioceptive Sensations. PLoS ONE.

[19] Ferrè E.R., Day B.L., Bottini G., Haggard P. (2013). How the vestibular system interacts with somatosensory perception: A sham-controlled study with galvanic vestibular stimulation. Neurosci. Lett..

[20] Romberg G.V., Holst E.V., Doden W. (1951). Über Zusammenhänge zwischen Wahrnehmung und objektivem Geschehen bei Wechselstromreizung des Vestibularapparates und optokinetischer Pendelreizung der Retina. Pflüg. Arch. Für Gesamte Physiol. Menschen Tiere.

[21] Aw S.T., Todd M.J., Halmagyi G.M. (2006). Latency and Initiation of the Human Vestibuloocular Reflex to Pulsed Galvanic Stimulation. J. Neurophysiol..

[22] Schneider E., Glasauer S., Brandt T., Dieterich M. (2003). Nonlinear Nystagmus Processing Causes Torsional VOR Nonlinearity. Ann. N. Y. Acad. Sci..

[23] Shuren J., Hartley T., Heilman K.M. (1998). The Effects of Rotation on Spatial Attention. Neuropsychiatr. Neuropsychol. Behav. Neurol..

[24] Saj A., Honore J., Bernati T., Coello Y., Rousseaux M. (2005). Subjective Visual Vertical in Pitch and Roll in Right Hemispheric Stroke. Stroke.

[25] Kerkhoff G., Zoelch C. (1998). Disorders of visuospatial orientation in the frontal plane in patients with visual neglect following right or left parietal lesions. Exp. Brain Res..

[26] Rubens A.B. (1985). Caloric stimulation and unilateral visual neglect. Neurology.

[27] Cappa S., Sterzi R., Vallar G., Bisiach E. (1987). Remission of hemineglect and anosognosia during vestibular stimulation. Neuropsychologia.

